# Comparison of Postoperative Outcomes of Duhamel and Transanal Endorectal Pull-Through in Hirschsprung Disease: A Propensity Score Study

**DOI:** 10.3390/pediatric18020056

**Published:** 2026-04-13

**Authors:** Jiraporn Khorana, Juthamas Jenyongsak, Kanokkan Tepmalai, Sireekarn Chantakhow

**Affiliations:** 1 Division of Pediatric Surgery, Department of Surgery, Faculty of Medicine, Chiang Mai University, Chiang Mai 50200, Thailand; prae_38_21@hotmail.com (J.J.); kanokkan.t@cmu.ac.th (K.T.); sireekarn.chan@cmu.ac.th (S.C.); 2 Clinical Surgical Research Center, Department of Surgery, Faculty of Medicine, Chiang Mai University, Chiang Mai 50200, Thailand; 3 Center of Clinical Epidemiology and Clinical Statistic, Faculty of Medicine, Chiang Mai University, Chiang Mai 50200, Thailand; 4 Department of Biomedical Informatics and Clinical Epidemiology, Faculty of Medicine, Chiangmai University, Chiang Mai 50200, Thailand

**Keywords:** hirschsprung disease, Duhamel, transanal endorectal pull-through (TERPT)

## Abstract

Background/Objectives: Hirschsprung disease (HSCR) is a congenital condition characterized by absence of ganglion cells in the distal bowel. The principle of surgical treatment is resection of the aganglionic bowel with restoration of intestinal continuity. Several operative techniques have been developed. This study aimed to compare outcomes between the Duhamel procedure and transanal endorectal pull-through (TERPT) in Hirschsprung disease using propensity score-based methods. Methods: Hirschsprung patients who underwent Duhamel or TERPT from January 2006 to December 2021 were included. The primary outcome was a composite endpoint at 6 months comprising obstructive symptoms, fecal soiling, or Hirschsprung-associated enterocolitis. Propensity scores were estimated via logistic regression incorporating eight preoperative covariates. The primary analysis employed overlap weighting (ATO), with multiple sensitivity analyses performed to assess robustness. Results: A total of 239 patients were included (TERPT, *n* = 181; Duhamel, *n* = 58). Before weighting, seven of eight covariates demonstrated meaningful imbalance (SMD > 0.10); ATO weighting achieved satisfactory balance across all covariates (all SMD < 0.10). A good composite outcome was achieved in 51.9% of TERPT and 53.4% of Duhamel patients, with no significant difference in the primary ATO-weighted analysis (OR 0.94, 95% CI 0.39–2.28; *p* = 0.897). No significant differences were observed in individual outcome components. Findings were consistent across all sensitivity analyses. TERPT was associated with significantly shorter operative time, lower estimated blood loss, and shorter hospital stay (all *p* < 0.001). Conclusions: No statistically significant differences were detected in 6-month postoperative functional outcomes between TERPT and the Duhamel operation. TERPT was associated with improved perioperative outcomes. However, these findings should be interpreted with caution due to limited statistical power and baseline differences between groups. Prospective multicenter studies with standardized outcome definitions and longer follow-up are warranted.

## 1. Introduction

Hirschsprung disease (HSCR) is a congenital condition characterized by the absence of ganglion cells in the distal bowel, resulting in impaired peristalsis of the affected segment and functional obstruction. Its incidence is approximately 1 in 5000 live births. Typical presentations are neonatal gut obstruction, constipation and enterocolitis [1,2]. Diagnosis is based on clinical evaluation, plain abdominal radiography, contrast enema, and histopathological confirmation [3,4,5]. The principle of surgical treatment is resection of the aganglionic bowel with restoration of intestinal continuity using normally innervated bowel while preserving anal sphincter function [6].

Several operative techniques have been developed, including the Swenson [7], Soave [8], Duhamel procedures [9] and the transanal endorectal pull-through (TERPT) [10]. The Duhamel operation has been widely used and is considered a reliable approach, particularly in patients with complex disease or significant colonic dilation. More recently, TERPT has gained popularity due to its minimally invasive nature and avoidance of abdominal incisions in many cases [11,12,13,14]. Despite advances in surgical technique and perioperative care, patients still experience postoperative morbidities such as obstructive symptoms, fecal soiling, and Hirschsprung-associated enterocolitis (HAEC) [15,16]. Comparative studies have reported variable outcomes. The Duhamel procedure has been associated with lower rates of postoperative enterocolitis, while TERPT offers advantages such as shorter operative time, reduced hospital stays, and faster recovery [17,18]. However, evidence regarding long-term outcomes remains limited and sometimes conflicting.

TERPT and open Duhamel are performed most frequently at many centers. Direct comparison of these techniques in observational studies is challenging because surgical approach selection is influenced by patient age, disease extent, preoperative condition, and evolving institutional practice over time. As a result, unadjusted comparisons are prone to confounding by indication. Propensity score methods offer a robust framework to address these biases and approximate causal inference in retrospective surgical cohorts. This study aims to compare 6-month postoperative outcomes between the Duhamel operation and TERPT in children with Hirschsprung disease using multiple propensity score-based analytical approaches.

## 2. Materials and Methods

### 2.1. Study Design and Setting

We conducted a retrospective cohort study of nonrandomized therapeutic outcomes at Chiang Mai University Hospital from January 2006 to December 2021. Data were obtained from electronic medical records of patients with HSCR who had at least 6 months of follow-up. The study was approved by the Chiang Mai University, Faculty of Medicine Ethics Committee (STUDY CODE: SUR-2564-08278; Research ID 8278). Informed consent was waived due to the retrospective design.

### 2.2. Participants

**Figure 1:**
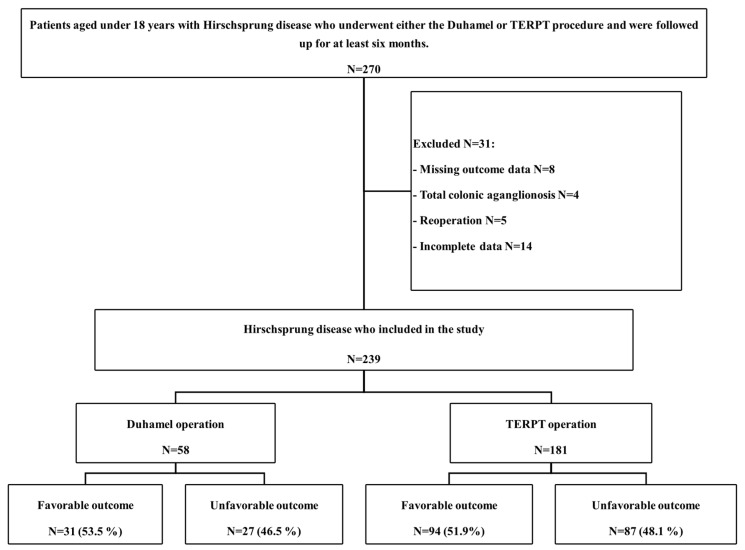
Study flow diagram.

Patients younger than 18 years with HSCR who underwent a Duhamel procedure or TERPT who postoperatively followed up for more than 6 months were included. Exclusion criteria were missing outcome data, total colonic aganglionosis, reoperation, or otherwise incomplete records. Among the five excluded patients who had undergone reoperation, three had reoperation due to retained aganglionic segments resulting from the absence of intraoperative frozen section at the primary institution or frozen section misinterpretation, and two had Hirschsprung disease diagnosed following initial surgery for anorectal malformation. Patients with Hirschsprung disease included in the study are shown in Figure 1.

### 2.3. Variables

Patient demographic and clinical data, including gender (male, female), gestational age (term, preterm, or unknown), age at surgery, and the presence of underlying diseases (e.g., Down syndrome, congenital heart, neurologic, or respiratory disease) were collected. Preoperative status was assessed via colostomy history and suspected HAEC. HAEC was diagnosed based on clinical presentations involving at least one symptom: vomiting, explosive diarrhea (with or without foul-smelling stools), abdominal distension, fever, or leukocytosis, supplemented by radiographic findings when available. Additionally, the transition-zone level on contrast enema was categorized ranging from ultrashort, rectum, rectosigmoid, sigmoid, descending colon, transverse colon and no transitional zone or noted as not applicable. We also divided the surgical era into three distinct periods (2006–2010, 2011–2015, and 2016–2021) for analysis.

### 2.4. Outcomes

The primary long-term composite outcome was classified as favorable (absence of obstructive symptoms, HAEC, and fecal soiling) or unfavorable (presence of any of these) and was assessed at ≥6 months postoperatively. Obstructive symptoms were defined as constipation and bloating; constipation was graded as follows: grade 1, intermittent and diet-responsive; grade 2, persistent and requiring laxatives; grade 3, refractory to diet and laxatives. HAEC was defined by fever, diarrhea, and abdominal distension. Fecal soiling was defined as inability to distinguish stool from gas and graded as: grade 1, one to two episodes per week; grade 2, daily without social impact; and grade 3, daily with social impact. Any grade ≥ 1 was considered soiling. Secondary outcomes were operative time, estimated blood loss (EBL) and length of hospital stay.

### 2.5. Treatment Groups

The two treatment arms were the Duhamel procedure and TERPT. The operative techniques were described in Supplementary File S1. In this retrospective study, procedure selection depended on surgeon preference. The Duhamel operation was predominantly performed during the earlier study period (2006–2010) and was preferentially selected for older patients and those with a prior diverting colostomy, whereas TERPT was progressively adopted thereafter and became the predominant technique from 2011 onward as institutional experience accumulated.

### 2.6. Sample Size

Prior to data collection, a sample size calculation was performed based on the rates of Hirschsprung-associated enterocolitis reported by Tannuri et al. [19], in which proportions of 3.4% and 20.0% were observed following the Duhamel and TERPT procedures, respectively. Using a two-proportion comparison with a two-sided alpha of 0.05 and 80% power, the estimated required sample size was 140 patients (70 per group).

### 2.7. Statistical Analysis

Statistical analysis was performed using STATA version 18 (StataCorp, Lakeway, TX, USA). Categorical descriptive data were reported as numbers and percentages, and the univariable analysis was performed with Fisher’s exact test. Numerical descriptive data were reported as mean and standard deviation or median and interquartile range, and univariable analysis was performed by using Student’s *t*-test or Mann–Whitney U-test depending on the distribution of data. Multivariable data was analyzed with logistic regression and reported in odds ratio.

Due to significant baseline imbalances across treatment groups, overlap weighting (ATO) was employed as the primary propensity score (PS) methodology to balance the covariates between the Duhamel and TERPT groups. This approach was chosen to maximize precision and minimize the impact of extreme weights. Propensity scores were calculated via logistic regression, incorporating covariates which were age at surgery, gender, gestational age, underlying disease, preoperative colostomy, suspected preoperative HAEC, transition zone level, and surgical era.

To ensure the robustness of our findings, sensitivity analyses were conducted using multiple approaches, including a Naïve (unadjusted) model, multivariable adjustment, propensity score matching, and various weighting techniques, such as Average Treatment Effect (ATE), Average Treatment Effect with Trimming (ATE with TR), and Average Treatment Effect on the Treated with Stabilized Weights (ATT with SW). Balance was assessed using the Standardized Mean Difference (SMD), where an absolute SMD of less than 10% indicated a negligible difference between groups.

Missing data were handled using complete case analysis, whereby only patients with complete data on all covariates included in the propensity score model and the primary outcome were eligible for the primary analysis.

## 3. Results

Between January 2006 and December 2021, a total of 270 patients were eligible for the study. Thirty-one patients were excluded due to missing outcome data, total colonic aganglionosis, had reoperation and incomplete data as shown in Figure 1. Consequently, 239 patients met the inclusion criteria and were enrolled in this study. The cohort was divided into two groups: the Duhamel group (*n* = 58) and the TERPT group (*n* = 181). Regarding clinical outcomes, favorable results were observed in 53.5% (*n* = 31) of the Duhamel group and 51.9% (*n* = 94) of the TERPT group.

The median age at surgery was 4.8 months (range, 2.5–17.6). There were 177 males (74.1%). Term birth was documented in 188 cases (78.7%), preterm birth in 25 cases (10.5%), and the remaining cases had unknown gestational status. Forty-six patients (19.3%) had underlying conditions. Preoperative colostomy was present in 67 patients (28.0%). Preoperative HAEC was identified in 20 patients (8.4%). The most frequent location of the transitional zone was the rectosigmoid junction, accounting for 132 patients (55.2%).

**Figure 2:**
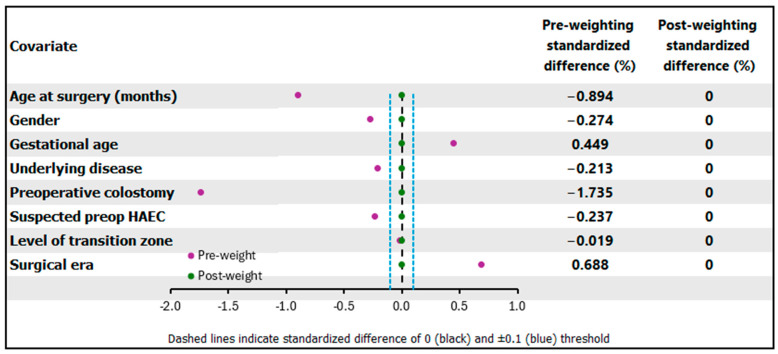
Covariate balance before and after overlap weighting (ATO).

Comparative baseline characteristics of the two procedures are shown in Table 1. The Duhamel group was older at diagnosis, had a higher rate of preoperative colostomy, and was more frequently performed in the earlier era compared with the TERPT group. Initially, several covariates showed imbalances between the two surgical groups as indicated by the pre-weighting SMD. However, after applying ATO, all baseline characteristics achieved perfect balance, indicating that the groups were well-balanced for subsequent outcome analysis (Figure 2).

**Figure 3:**
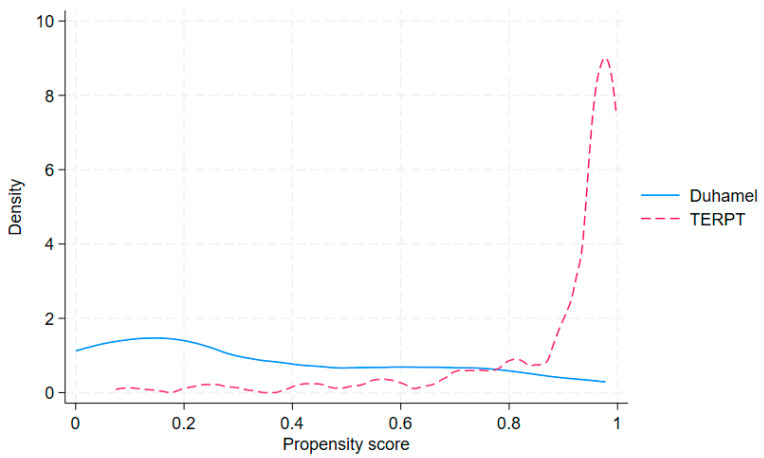
Propensity score distribution by treatment group before weighting by Kernel density plot.

Figure 3 illustrates the distribution of estimated propensity scores. The Duhamel group (solid blue line) showed a uniform distribution across the 0–0.8 range, while the TERPT group (dashed pink line) was markedly right-skewed, concentrating near 1.0. This limited overlap at the extremes indicates significant selection bias and justifies the use of overlap weighting (ATO) to focus inference on patients within the region of common support.

**Figure 4:**
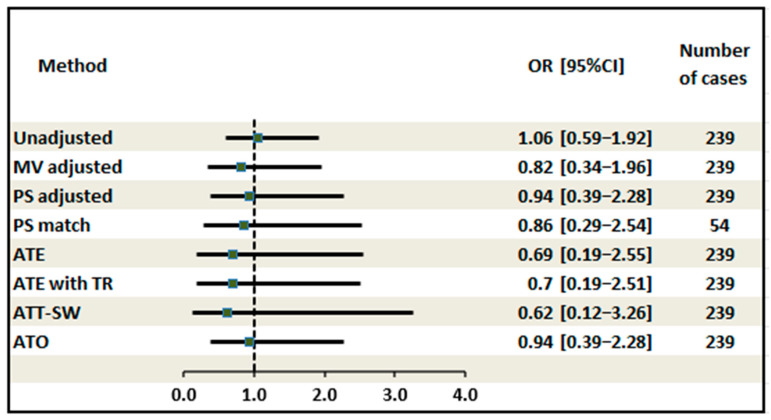
Forest plot: odds ratio across all sensitivity analysis methods. Note: Multivariable adjusted for age at surgery, gender, gestational age, underlying diseases, preoperative colostomy, suspected preoperative HAEC, transition zone level, and surgical era. Propensity scores calculated via logistic regression incorporating the same preoperative covariates. Weighting models used to balance baseline imbalances between groups. Odds ratios > 1 indicate a higher probability of a favorable outcome with the TERPT procedure compared to the Duhamel procedure. Abbreviations: MV: multivariable; PS: propensity scores; ATE: average treatment effect; ATE with TR: average treatment effect with trimming; ATT with SW: average treatment effect on the treated with stabilized weights; and ATO: overlap weighting.

Before weighing, seven out of eight covariates had an absolute SMD exceeding 0.10, indicating meaningful baseline imbalance. Following application of ATO, all covariates achieved an absolute SMD below 0.10, indicating satisfactory balance (Figure 3). The effective sample sizes after ATO weighting were 19.9 for the TERPT group (out of 181 patients; 11%) and 32.9 for the Duhamel group (out of 58 patients; 57%), suggesting substantial down-weighting particularly in the TERPT group. This finding is consistent with the observed limited overlap between the two treatment groups across the propensity score distribution (Figure 3), wherein a large proportion of TERPT patients had propensity scores in regions with sparse Duhamel representation. Given the low effective sample size, results from the ATO-weighted analysis should be interpreted with caution, and findings from multiple sensitivity analyses are presented in Figure 4 to assess the robustness of the primary estimates.

Postoperative outcomes and perioperative parameters are summarized in Table 2. At 6 months postoperatively, the favorable composite outcome had no statistically significant difference between the two procedures (OR 0.94, 95% CI 0.39–2.28; *p* = 0.897). With respect to individual outcome components, HAEC, obstructive symptoms and fecal soiling, none of these reached statistical significance. In contrast, significant differences were observed in perioperative parameters. The TERPT procedure was associated with a shorter operative time, lower estimated blood loss and shorter length of hospital stay compared with the Duhamel operation. Notably, the median follow-up duration was significantly longer in the TERPT group than in the Duhamel group, reflecting the historical transition in operative technique at this institution over the study period.

Figure 4 presents the results of multivariable and propensity score-based sensitivity analyses examining the odds of a favorable composite outcome at 6 months for TERPT compared with the Duhamel procedure. Across all analytic approaches, TERPT was not associated with a statistically significant difference in the composite outcome compared with the Duhamel operation. In the naïve unadjusted analysis, the odds ratio (OR) for a favorable outcome with TERPT was 1.06 (95% CI 0.59–1.92; *p* = 0.841). After multivariable adjustment for age at surgery, sex, gestational age, underlying disease, preoperative colostomy, suspected preoperative HAEC, transitional zone level, and surgical era, the OR was 0.82 (95% CI 0.34–1.96; *p* = 0.652). Propensity score adjustment and matching yielded consistent estimates (OR 0.94, 95% CI 0.39–2.28 and OR 0.86, 95% CI 0.29–2.54, respectively). Among weighting-based approaches, the ATE, ATE with trimming, and ATT with stabilized weights produced OR estimates ranging from 0.62 to 0.70, with overlapping confidence intervals and *p*-values exceeding 0.57 in all cases. The primary analysis using overlap weighting (ATO) yielded an OR of 0.94 (95% CI 0.39–2.28; *p* = 0.897). The direction and magnitude of effect estimates were broadly consistent across all methods, supporting the robustness of the primary finding of no significant difference in favorable outcomes between the two operative techniques.

## 4. Discussion

This single-center retrospective cohort study compared postoperative outcomes between TERPT and the Duhamel operation in pediatric patients with Hirschsprung disease, using propensity score-based methods to account for measured confounding. In our cohort, the Duhamel operation was preferentially chosen for patients with a prior colostomy and those who were older at the time of surgery, in whom minimizing rectal dissection was considered advantageous. In the latter part of the study period, TERPT became increasingly favored due to the absence of a residual rectal pouch and growing institutional experience with the technique. The principal finding of this study is that TERPT and the Duhamel operation were not associated with a statistically significant difference in the composite outcome of obstructive symptom, fecal soiling, or Hirschsprung-associated enterocolitis at 6 months postoperatively, with approximately half of patients in both groups achieving a good outcome (51.9% vs 53.4%, respectively). This finding was consistent in direction and magnitude across all analytic approaches examined in the sensitivity analysis, including multivariable adjustment, propensity score adjustment, PS matching, and multiple weighting strategies, thereby supporting the robustness of our primary estimate. In contrast, TERPT was associated with significantly superior perioperative outcomes, including shorter operative time, lower estimated blood loss, and shorter length of hospital stay, findings that align with the proposed technical advantages of the transanal approach [14].

The absence of a significant difference in postoperative functional outcomes between TERPT and Duhamel in our study is generally consistent with previous comparative studies and systematic reviews, although some differences have been reported. A systematic review by Mao et al. of six studies (*n* = 280) found no difference in fecal incontinence or operative time, while TERPT was associated with a shorter hospital stay and a higher rate of enterocolitis; however, substantial heterogeneity was noted, and larger studies with longer follow-up were recommended [18]. Duhamel tended to be performed in older patients, likely related to the need for a wider rectal lumen to accommodate stapling for the side-to-side anastomosis which may also explain the higher frequency of preoperative colostomy in that group. In older patients, pelvic dissection can be associated with increased blood loss. The prior literature is mixed: for example, Allin et al. reported a lower incidence of fecal incontinence after Duhamel compared with another technique [20]. Postoperative complications after Hirschsprung surgery have been reported in up to 60% of patients [21,22]. In our cohort, 47.7% of patients experienced unfavorable outcomes at 6 months postoperatively; however, after propensity score adjustment, complication rates remained comparable between groups. TERPT showed a nonsignificant trend toward better overall outcomes. Our finding of longer operative time and length of hospital stay after Duhamel aligns with the larger meta-analysis by Wang et al. [23].

In our study, the composite outcome included fecal soiling, which is only clinically assessable following the completion of toilet training, typically expected by 2 years of age. The soiling component of the composite outcome may have been subject to misclassification in patients who had not yet completed toilet training at the time of the 6-month postoperative assessment. Supplementary File S2 provides the combined analysis, pooling estimates across age subgroups using a clustered approach to account for the correlation between patients aged less than and more than 2 years, which reflects their toilet training status.

A key methodological strength of this study is the application of propensity score-based methods to address the substantial baseline imbalances inherent in this retrospective cohort, most notably the tendency for older patients and those operated during earlier surgical eras to have undergone the Duhamel procedure. The ATO estimand explicitly targets inference toward the population of patients for whom genuine clinical equipoise between the two procedures exists, which represents the most clinically relevant comparison in this context. However, the low effective sample size after ATO weighting particularly in the TERPT group (Effective sample size-ESS from 181 patients) reflects the limited overlap between groups and should be acknowledged when interpreting the precision of effect estimates.

Several limitations of this study warrant careful consideration. First, as a single-center retrospective study, our findings may not be generalizable to other institutions. Second, despite the use of propensity score weighting to balance measured confounders, unmeasured confounding such as surgeon experience, intraoperative findings, individual anatomical variation, or family compliance with postoperative care cannot be excluded, and no formal sensitivity analysis for hidden bias was performed. Third, the composite outcome was assessed at 6 months postoperatively; given that bowel function in HSCR patients may continue to evolve over years, longer-term follow-up data would be required to fully characterize the trajectory of outcomes for each technique. Fourth, the substantially longer median follow-up in the TERPT group (69 vs. 27.5 months) may introduce differential surveillance bias, as patients with longer follow-up have greater opportunity for outcome detection, potentially inflating outcome rates in the TERPT group. Fifth, the sample size calculation was based on previously reported rates of enterocolitis alone, whereas the primary outcome in this study was a composite endpoint with observed event rates substantially closer between groups than assumed. Combined with the reduced effective sample size after overlap weighting, the study was likely underpowered to detect a clinically meaningful difference in the composite outcome, and the non-significant findings should not be interpreted as evidence of equivalence between the two procedures. Finally, the limited overlap in propensity score distributions between the two groups resulted in a low effective sample size after ATO weighting, which reduces the statistical power of the primary analysis and widens confidence intervals, precluding definitive conclusions regarding equivalence or superiority of either technique.

Given the absence of statistically significant differences in functional outcomes, procedure selection may be individualized based on patient characteristics and surgeon expertise, while acknowledging the limitations of the current study.

## 5. Conclusions

This propensity score-weighted retrospective cohort study found no statistically significant difference in postoperative enterocolitis, obstructive symptoms, or fecal soiling between TERPT and the Duhamel operation at 6 months postoperatively. TERPT was associated with significantly shorter operative time, lower estimated blood loss, and shorter length of hospital stay. The consistency of findings across multiple sensitivity analyses supports the robustness of these results. Nevertheless, given the limited effective sample size after overlap weighting and the wide confidence intervals observed, the study was insufficiently powered to establish equivalence, and a clinically meaningful difference between the two techniques cannot be excluded. Prospective multicenter studies with adequate sample sizes and standardized outcome definitions are warranted to provide more definitive evidence.

## Figures and Tables

**Table 1:** Baseline characteristics with *p*-values and SMD (pre/post weight).

Characteristics	Duhamel Operation *n* = 58	TERPT Operation *n* = 181	*p*-Value	SMD (Pre)	SMD (Post)
Age at surgery (months)	22.12 (11.70–43.67)	3.47 (2.23–6.90)	<0.001	−0.894	0.000
Gender			0.088	−0.274	0.000
Female	10 (17.2%)	52 (28.7%)			
Male	48 (82.8%)	129 (71.3%)			
Gestational age			<0.001	0.449	0.000
Term	5 (8.6%)	20 (11.0%)			
Preterm	38 (65.5%)	150 (82.9%)			
Unknown	15 (25.9%)	11 (6.1%)			
Underlying disease	15 (25.9%)	31 (17.1%)	0.179	−0.213	0.000
Preoperative colostomy	45 (77.6%)	22 (12.2%)	<0.001	−1.735	0.000
Suspected preop HAEC	8 (13.8%)	12 (6.6%)	0.103	−0.237	0.000
Level of transition zone			0.190	−0.019	0.000
NA	3 (5.2%)	2 (1.1%)			
Ultrashort	4 (6.9%)	5 (2.8%)			
Rectum	10 (17.2%)	32 (17.7%)			
Rectosigmoid	26 (44.8%)	106 (58.6%)			
Sigmoid	5 (8.6%)	14 (7.7%)			
Descending colon	1 (1.7%)	3 (1.7%)			
Transverse colon	2 (3.4%)	2 (1.1%)			
No T zone	7 (12.1%)	17 (9.4%)			
Surgical era			<0.001	0.688	0.000
2006–2010	35 (60.3%)	43 (23.8%)			
2011–2015	13 (22.4%)	78 (43.1%)			
2016–2021	10 (17.2%)	60 (33.1%)			

*p*-values are calculated using the Mann–Whitney U test for continuous variables and Fisher’s Exact test for categorical variables. SMD (pre/post) represents the Standardized Mean Difference before and after applying propensity score weighting with ATO (overlap weighting). Age in months is calculated as age in days divided by 30. Abbreviations: HAEC: Hirschsprung-associated enterocolitis; SMD: standardized mean difference; and TERPT: transanal endorectal pull-through; NA: not available (not documented in the medical record).

**Table 2:** Postoperative overall outcome and perioperative parameters.

Characteristic	Duhamel Procedure *n* = 58	TERPT Procedure *n* = 181	Odds Ratio (95% CI)	*p*-Value
Composite outcome			0.94 (0.39–2.28)	0.897
Favorable	31 (53.4%)	94 (51.9%)		
Unfavorable	27 (46.6%)	87 (48.1%)		
Enterocolitis	16 (27.6%)	44 (24.3%)	1.17 (0.43–3.15)	0.762
Obstructive symptom	21 (36.2%)	54 (29.8%)	0.50 (0.20–1.25)	0.139
Fecal soiling	10 (17.2%)	34 (18.8%)	1.17 (0.38–3.62)	0.784
Operative time (hours)	3.3 (1.0)	2.0 (0.9)	NA	<0.001 *
Estimate blood loss (ml)	20 (15–50)	5 (2–10)	NA	<0.001 ^#^
Length of stay (days)	11 (10–18)	9 (7–14)	NA	<0.001 ^#^
Follow up time (months)	27.5 (11–94)	69 (31–98)	NA	<0.001 ^#^

* *p*-value from Student’s *t*-test; ^#^ *p*-value from Mann–Whitney U test. Abbreviations: 95% CI: 95% confidence interval; and NA: not applicable.

## Data Availability

Dataset available from the corresponding author at jiraporn.kho@cmu.ac.th.

## Supplementary Materials

The following supporting information can be downloaded at: https://www.mdpi.com/article/10.3390/pediatric18020056/s1, Supplementary File S1. The operative techniques of Duhamel’s procedure and Transanal endorectal pull-through; Supplementary File S2. The combined analysis, pooling estimates across age subgroups using a clustered approach to account for the correlation between patients aged less than and more than 2 years, reflects their toilet training status.

## Abbreviations

The following abbreviations are used in this manuscript:

HSCR	Hirschsprung disease
TERPT	Transanal endorectal pull-through
HAEC	Hirschsprung-associated enterocolitis
EBL	Estimated blood loss
PS	Propensity score
ATO	Overlap weighting
ATE	Average treatment effect
ATE with TR	Average treatment effect with trimming
ATT with SW	Average treatment effect on the treated with stabilized weights
SMD	Standardized mean difference
OR	Odds ratio
95% CI	95% confidence interval
MV	Multivariable
