# Supplementary material for: Comparison of Postoperative Outcomes of Duhamel and Transanal Endorectal Pull-Through in Hirschsprung Disease: A Propensity Score Study
Source: Pediatr Rep. 2026 Apr 13;18(2):56. doi: 10.3390/pediatric18020056 (PMC13118591; doi:10.3390/pediatric18020056)
Supplement: Supplementary file 1 [file pediatrrep-18-00056-s001.zip › pediatrrep-4239687-Supplementary File 1 Operative technique.pdf]

Supplementary File 1

Operative technique

Duhamel's Procedure

Abdominal phase (open or laparoscopic-assisted)

- Explore abdomen: identify transition zone; take seromuscular or full-thickness "leveling" biopsies until ganglionated bowel confirmed.
- Colon Mobilization: The healthy, normal colon is carefully dissected and freed from its surrounding attachments. The aganglionic section of the rectum is then cut and its end is closed off.
- Retrorectal Tunnel Creation: A tunnel is meticulously created behind the aganglionic rectum, in a plane free of blood vessels, to serve as a conduit for the healthy colon.

Perineal phase

- Rectal incision: Make a low posterior rectal wall incision (typically 1–2 cm above the dentate line), entering the rectal lumen.
- Colon Pull-Through: A long clamp is guided through the anus and into the retrorectal tunnel. It grasps the healthy colon and pulls it down to exit through the anus.
- Trimming: The excess portion of the pulled-through colon is trimmed off, and the remaining healthy colon is secured to the anus with sutures.
- Anastomosis and Stapling: The pulled-through healthy colon is laid side-by-side with the aganglionic rectum. A surgical stapler is inserted through the anus to create a wide side-to-side anastomosis by cutting the common wall between the two segments. This forms a new, single passage for stools.

Abdominal phase (Closure)

- Trimming the rectal pouch: Excess rectal pouch was trimmed near the stapler line to prevent formation of a blind pouch; closure was achieved either with a linear stapler or with hand-sewn sutures.
- Closure of the abdominal incision

Transanal endorectal pull-through (TERPT)

- Transanal exposure: Place an anal retractor and stay sutures to evert the anal canal. Make a circumferential mucosal incision 0.5–1 cm above the dentate line.
- Submucosal dissection: Perform submucosal dissection proximally to create a short muscular cuff of rectum (Soave-type). Keep the cuff short. If a longer cuff was created, consider posterior myotomy/myectomy or partial cuff division.
- Proximal mobilization & leveling biopsies: Advance dissection cephalad within the submucosal plane until the rectum is free; then continue on the colon wall. Obtain sequential seromuscular "leveling" biopsies to pass the transition zone and confirm

ganglionated bowel by frozen section. If needed, use laparotomy or laparoscopic assistance for proximal mobilization, vessel control, and additional biopsies.

- Pull-through:
  Deliver the ganglionated colon transanally through the muscular cuff. Avoid twist; check that reach is tension-free.
- Division of aganglionic segment: Divide the aganglionic colon at the confirmed level, ensuring adequate perfusion and length of the ganglionated pull-through segment.
- Anastomosis (Soave-type coloanal): Fashion a low hand-sewn coloanal anastomosis between the pulled-through ganglionated bowel and the anoderm/rectal cuff mucosa. Ensure a wide lumen and good perfusion.

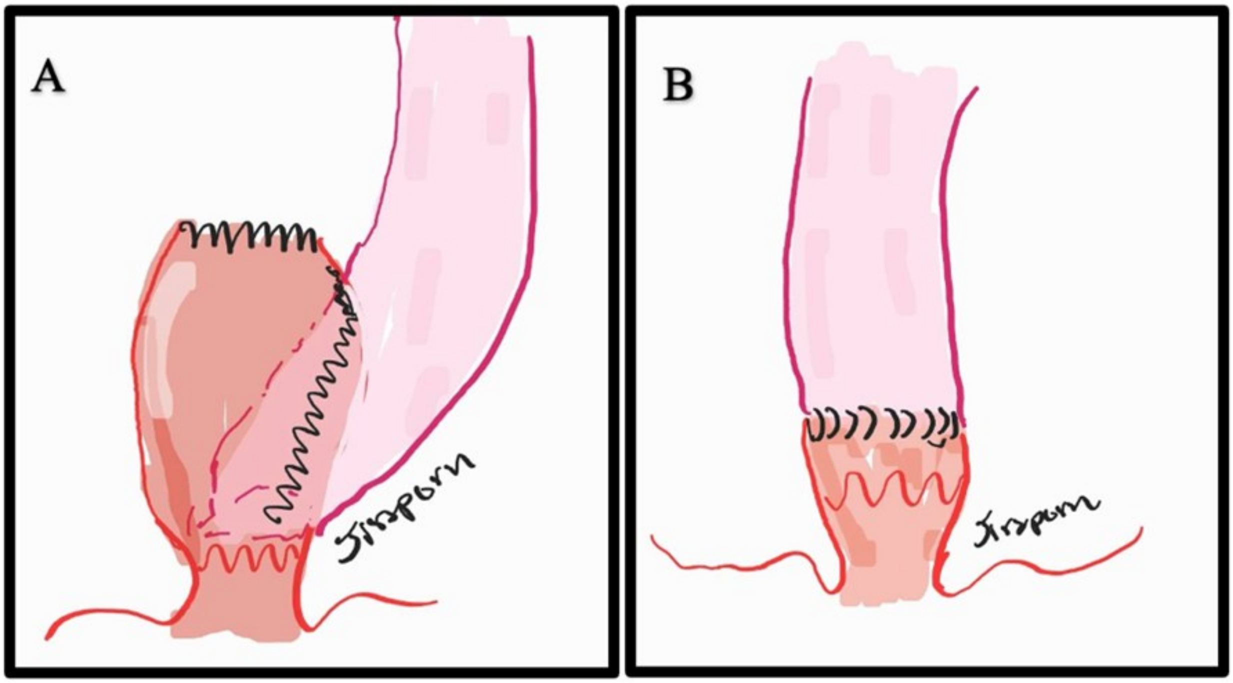

Figure A showed the anastomosis technique of Duhamel’s operations.

Figure B showed the anastomosis technique of Transanal endorectal pull-through.
