# Supplementary material for: Comparison of Postoperative Outcomes of Duhamel and Transanal Endorectal Pull-Through in Hirschsprung Disease: A Propensity Score Study
Source: Pediatr Rep. 2026 Apr 13;18(2):56. doi: 10.3390/pediatric18020056 (PMC13118591; doi:10.3390/pediatric18020056)

The combined analysis, pooling estimates across age subgroups using a clustered approach to account for the correlation between patients aged less than and more than 2 years, reflects their toilet training status.

<table>
<tr><th rowspan="2">Outcome</th><th>Total cohort N=239</th><th rowspan="2">p-value</th><th>Age more than 2 years N=58</th><th rowspan="2">p-value</th><th>Age less than 2 years N=181</th><th>p-value</th></tr>
<tr><th>Odds ratio (95% Confidence Interval)</th><th>Odds ratio (95% Confidence Interval)</th><th>Odds ratio (95% Confidence Interval)</th><th></th></tr>
<tr><td>Composite</td><td>0.94 (0.39-2.28)</td><td>0.897</td><td rowspan="2">0.71 (0.17-2.98)</td><td rowspan="2">0.635</td><td rowspan="2">1.26 (0.38-4.21)</td><td rowspan="2">0.703</td></tr>
<tr><td>Combine 2-groups</td><td>0.94 (0.53-1.66)</td><td>0.840</td></tr>
<tr><td>Enterocolitis</td><td>1.17 (0.43-3.15)</td><td>0.762</td><td rowspan="2">2.29 (0.50-10.63)</td><td rowspan="2">0.288</td><td rowspan="2">0.70 (0.18-2.65)</td><td rowspan="2">0.596</td></tr>
<tr><td>Combine 2-groups</td><td>1.17 (0.38-3.62)</td><td>0.790</td></tr>
<tr><td>Obstructive symptom</td><td>0.50 (0.20-1.25)</td><td>0.139</td><td rowspan="2">0.46 (0.10-1.99)</td><td rowspan="2">0.297</td><td rowspan="2">0.64 (0.18-2.27)</td><td rowspan="2">0.488</td></tr>
<tr><td>Combine 2-groups</td><td>0.50 (0.36-0.69)</td><td><0.001</td></tr>
<tr><td>Fecal soiling</td><td>1.17 (0.38-3.62)</td><td>0.784</td><td rowspan="2">0.62 (0.10-3.79)</td><td rowspan="2">0.607</td><td rowspan="2">6.20 (0.73-52.54)</td><td rowspan="2">0.094</td></tr>
<tr><td>Combine 2-groups</td><td>1.17 (0.22-6.27)</td><td>0.854</td></tr>
</table>

The combined analysis of obstructive symptoms demonstrated a statistically significant reduction associated with TERPT compared with the Duhamel operation (OR 0.50, 95% CI 0.36–0.69; $p<0.001$), a finding consistent across both age subgroups (age ≥2 years: OR 0.46, 95% CI 0.10–1.99; age <2 years: OR 0.64, 95% CI 0.18–2.27), though neither subgroup alone reached statistical significance, likely reflecting limited power within individual strata. These findings suggest that the perioperative and functional outcome comparisons in our primary analysis were not substantially driven by differential toilet training status between groups.

Among patients aged less than 2 years at surgery, fecal soiling at 6 months was numerically more frequent in the TERPT group compared with the Duhamel group (OR 6.20, 95% CI 0.73–52.54; $p=0.094$). However, this estimate should be interpreted with considerable caution given the very wide confidence interval, reflecting a small number of events within this subgroup, and the finding did not reach statistical significance.

Outcome Distribution Chart in patient who age less that 24 months

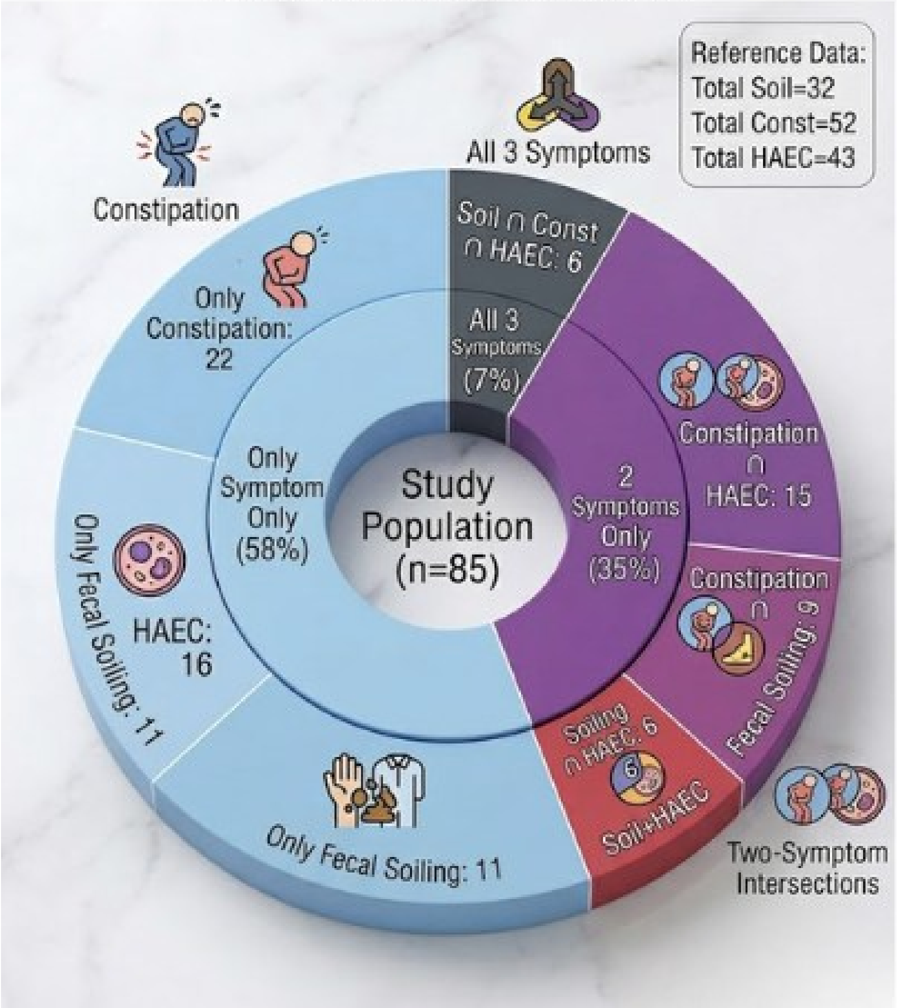

Supplement: Supplementary file 1 [file pediatrrep-18-00056-s001.zip › pediatrrep-4239687-Supplementary File 2 Combined analysis.pdf]
